# Cutaneous reactions with enfortumab vedotin: A case series and review of the literature

**DOI:** 10.1016/j.jdcr.2021.05.020

**Published:** 2021-06-04

**Authors:** Allison S. Dobry, Cesar Antonio Virgen, Anna-Marie Hosking, Nataliya Mar, Linda Doan, Bonnie Lee, Janellen Smith

**Affiliations:** a Department of Dermatology, University of California, Irvine, California; b Department of Dermatology, Brigham and Women's Hospital, Boston, Massachusetts; c Division of Hematology/Oncology, University of California, Irvine, California

**Keywords:** enfortumab vedotin, drug eruption, drug reaction, oncology, oncologic therapy, ADC, antibody-drug conjugate, EV, enfortumab vedotin

## Introduction

Enfortumab vedotin (EV) is a novel treatment for patients with advanced urothelial carcinoma who have failed platinum-based chemotherapy and immunotherapy.^1^ EV is an antibody-drug conjugate (ADC) composed of a humanized anti-nectin-4 monoclonal antibody attached to a microtubule inhibitor.^2^ We report 3 individuals with unique cutaneous reactions after EV treatment.

### Case 1

**Fig 1:**
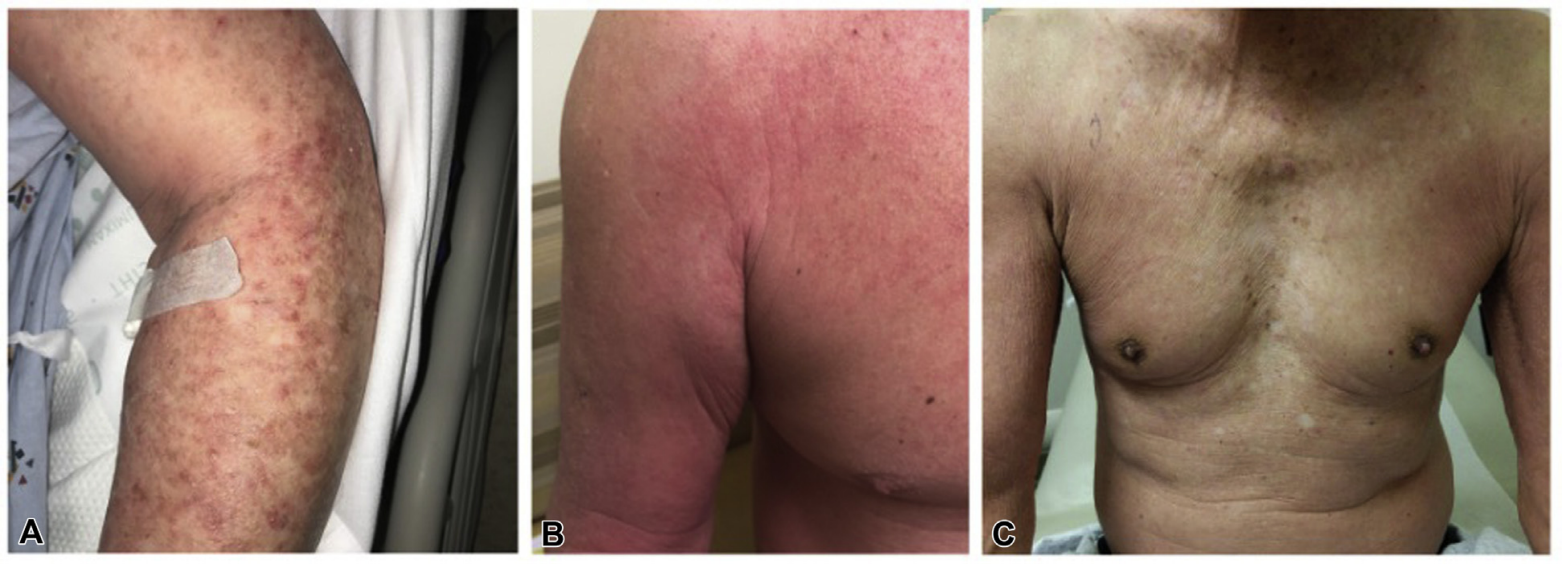
**A,** Case 1 presented with scattered ill-defined erythematous macules and papules with an associated scale on the upper portion of the chest, dorsal aspects of the arms, proximal portions of the thighs, and periocular crusted exudate. **B,** Case 2 presented with diffusely erythematous, indurated plaques on the upper portions of the arms, upper portion of the chest, and thighs. **C,** Case 3 presented with erythematous patches on the trunk, upper portion of the extremities, and upper medial portion of the thighs.

**Fig 2:**
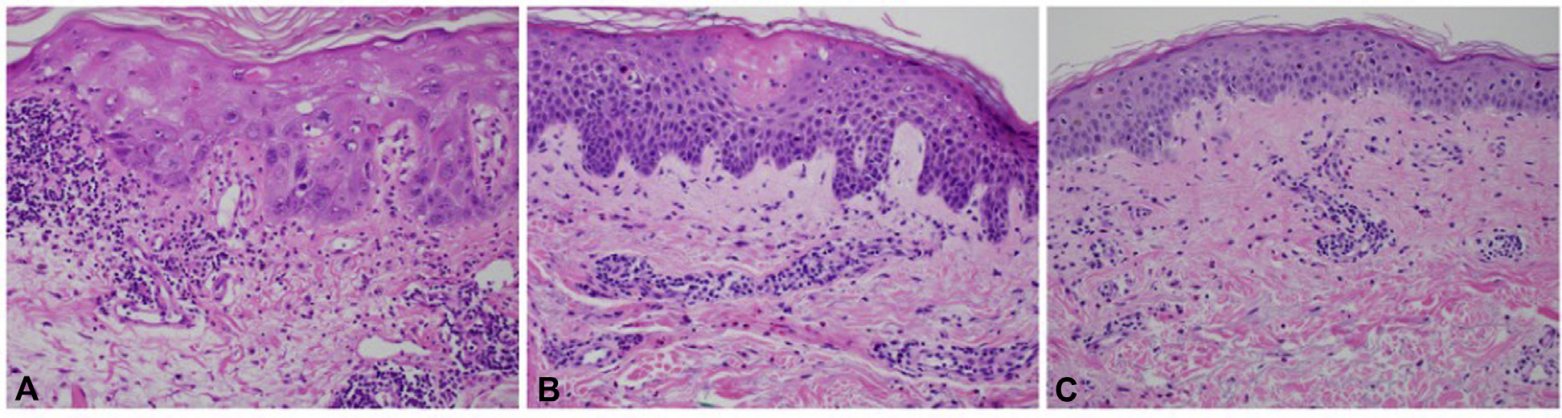
Histopathology of the 3 cases. **A,** Case 1 had marked dyskeratosis and epidermal dysmaturation with a sparse superficial perivascular infiltrate of lymphocytes and eosinophils and a subtle vacuolar interface. Biopsy location: left upper portion of the arm. **B,** Case 2 had spongiosis with epidermal necrosis and infiltrate with eosinophils. Biopsy location: right upper portion of the arm. **C,** Case 3 had epidermal atypia, apoptosis, and superficial perivascular dermatitis with eosinophils and focal interface change. Biopsy location: abdomen. (**A, B,** and **C,** Hematoxylin-eosin stain; original magnification: ′20.)

A 75-year-old man with stage IV urothelial cancer previously on carboplatin/gemcitabine and pembrolizumab presented with rash, hypotension, dyspnea, vomiting, and diarrhea 7 days after initiation of EV. On examination, he had scattered ill-defined scaly erythematous papules on the chest, arms, and thighs (Fig 1, *A*). Histopathology revealed subtle interface dermatitis accompanied by a perivascular lymphocytic infiltrate with eosinophils and neutrophils, marked dyskeratosis, and epidermal dysmaturation (Fig 2, *A*). Although the rash improved with clobetasol ointment, 16 days after EV infusion, he was transferred to the medical intensive care unit for vasodilatory shock concerning hypersensitivity reaction and received prednisone. He later developed renal failure, shock liver, and candidemia, and died 3 weeks after presentation.

### Case 2

A 65-year-old man with stage IV urothelial carcinoma on pembrolizumab (previously treated with cisplatin/gemcitabine, nivolumab, and pemetrexed) presented with a pruritic rash 13 days after EV infusion. The dermatologic examination was notable for diffusely erythematous, indurated plaques on the arms, chest, and thighs (Fig 1, *B*). Dermatopathology showed spongiosis with epidermal atypia, necrosis, and superficial perivascular infiltrate with eosinophils (Fig 2, *B*). His rash improved with triamcinolone 0.1% ointment. He was restarted on dose-reduced EV without rash recurrence.

### Case 3

A 77-year-old man with stage IV urothelial carcinoma developed a pruritic rash on the trunk and extremities 5 days after initiating treatment with EV, carboplatin, and pembrolizumab. Physical examination revealed erythematous patches on the trunk, arms, and thighs (Fig 1, *C*). Histopathology showed keratinocyte atypia, apoptosis, and superficial perivascular dermatitis with eosinophils and focal interface change (Fig 2, *C*). The rash resolved with prednisone and fluocinonide 0.05% ointment and did not recur with retreatment at standard dosing.

## Discussion

EV is an ADC approved as a third-line treatment of locally advanced or metastatic urothelial cancer. ADCs are novel targeted therapeutic agents comprised of a highly specific antibody against a tumor antigen linked to a cytotoxic payload that becomes activated upon drug internalization into the tumor cell. Nectin-4 is a cell adhesion molecule with increased expression in urothelial and breast cancers but is also expressed at low levels in keratinocytes, sweat glands, and hair follicles.^3^

Rashes are documented in 48% of trial patients receiving EV, typically occurring 15.9 days after initiation.^4^ Grade 3-4 skin reactions have been documented in 10% of patients, with diagnoses including symmetrical drug-related intertriginous and flexural exanthema, bullous dermatitis, exfoliative dermatitis, and palmar-plantar erythrodysesthesia.^5^ Two similar cases of papulosquamous eruptions with EV treatment have been reported.^6^^,^^7^ In the first, histopathology demonstrated vacuolar interface dermatitis with keratinocyte dysmaturation and lympho-eosinophilic infiltrate. In the second, histopathology revealed full-thickness epidermal necrosis with a lichenoid infiltrate. It is unclear whether the reactions observed in these patients are due to apoptosis in nectin-4-expressing keratinocytes or a hypersensitivity reaction to one of the components (possibly primed by antecedent/concurrent pembrolizumab treatment), or even a combination of these 2 mechanisms. In the review of these 3 cases and the previously reported cases, keratinocyte dysmaturation and apoptosis of the epidermis with either spongiosis or interface changes, in addition to a superficial perivascular infiltrate with eosinophils, appear to be characteristic features of EV drug eruption. All 3 patients experienced an improvement in their eruptions with corticosteroids, and 2 were able to continue EV therapy uninterrupted. It is vital that dermatologists familiarize themselves with novel cutaneous toxicities and maintain suspicion for cutaneous severe adverse events to facilitate oncologic therapy without interruption.

## Conflicts of interest

None disclosed.
